# The Society for Pediatric Anesthesia recommendations for the use of opioids in children during the perioperative period

**DOI:** 10.1111/pan.13639

**Published:** 2019-06-11

**Authors:** Joseph P. Cravero, Rita Agarwal, Charles Berde, Patrick Birmingham, Charles J. Coté, Jeffrey Galinkin, Lisa Isaac, Sabine Kost‐Byerly, David Krodel, Lynne Maxwell, Terri Voepel‐Lewis, Navil Sethna, Robert Wilder

**Affiliations:** ^1^ Department of Anesthesiology, Critical Care, and Pain Medicine Boston Children's Hospital, Harvard Medical School Boston Massachusetts; ^2^ Pediatric Anesthesiology Department Lucille Packard Children's Hospital, Stanford University Medical School Stanford California; ^3^ Department of Anesthesiology Ann and Robert H. Lurie Children's Hospital Northwestern University Feinberg School of Medicine Evanston Illinois; ^4^ Department of Anesthesiology Mass General Hospital for Children, Harvard University Boston Massachusetts; ^5^ Anesthesiology Department Children's Hospital of Colorado, University of Colorado Aurora Colorado; ^6^ Department of Anesthesia and Pain Medicine Hospital for Sick Children, University of Toronto Toronto Ontario Canada; ^7^ Pediatric Anesthesiology and Critical Care Medicine Johns Hopkins University Hospital Baltimore Maryland; ^8^ Department of Aneshtesiology and Critical Care Medicine Children's Hospital of Philadelphia, Perelman School of Medicine at the University of Pennsylvania Philadelphia; ^9^ Department of Aneshteiology C. S. Mott Children's Hospital, University of Michigan Medical School Ann Arbor Michigan; ^10^ Department of Anesthesiology and Perioperative Medicine Mayo Clinic Rochester Minnesota

**Keywords:** monitoring, opioids, patient‐controlled analgesia, recommendations, side effects

## Abstract

Opioids have long held a prominent role in the management of perioperative pain in adults and children. Published reports concerning the appropriate, and inappropriate, use of these medications in pediatric patients have appeared in various publications over the last 50 years. For this document, the Society for Pediatric Anesthesia appointed a taskforce to evaluate the available literature and formulate recommendations with respect to the most salient aspects of perioperative opioid administration in children. The recommendations are graded based on the strength of the available evidence, with consensus of the experts applied for those issues where evidence is not available. The goal of the recommendations was to address the most important issues concerning opioid administration to children after surgery, including appropriate assessment of pain, monitoring of patients on opioid therapy, opioid dosing considerations, side effects of opioid treatment, strategies for opioid delivery, and assessment of analgesic efficacy. Regular updates are planned with a re‐release of guidelines every 2 years.

## 1 introduction

The treatment of perioperative pain in children has been a topic of great interest to pediatricians, pediatric subspecialists, surgeons, and anesthesiologists for many years. It is part of our core mission to provide high quality and ethical care to pediatric patients in the perioperative time frame.^1^, ^2^, ^3^, ^4^, ^5^, ^6^ The role of opioid medications in the treatment of discomfort after surgery has been highlighted both as a critical component of adequate pain control and as the primary factor contributing to perioperative complications.^7^ Unfortunately, few evidence‐based reports are available to guide the use of opioid medications in children in the perioperative period.^8^ The evidence that exists is published in a wide variety of professional journals, newsletters, meeting proceedings, etc.^9^ Many of the recommendations that have been written concerning opioid administration address this topic as a small part of overall pain management and rarely address pediatric specific issues in detail.^10^ For example, a recently published set of recommendations regarding the management of postoperative pain covers many aspects of perioperative pain control, but does not address several important issues specific to the administration of opioids to children, such as nurse‐controlled analgesia (NCA), opioid contraindications in children, or special monitoring requirements in this age group.^10^

The Society for Pediatric Anesthesia (SPA) initiated and supported the formulation of these recommendations with the intent of providing guidance for pediatric anesthesiologists that is (where possible) evidence‐based or a synthesis of expert opinion where evidence is lacking. The authors did not attempt to make this manuscript all encompassing, but rather were interested in addressing the issues that were felt to be in need of clarification at this time. This document should not be interpreted as an endorsement of the primacy of opioids in the management of perioperative pain. Rather, this project begins with the admonition that clinicians should use all methods possible to minimize the use of opioids, including the administration of adjunctive systemic medications, regional anesthesia, and alternative medicine techniques.

## 2 definitions

This document addresses topics related to the use of opioid medications including methods of administration, assessment of pain, optimization of therapy, and monitoring of pediatric patients on opioid therapy. This discussion will largely be limited to pharmaceuticals available in North America; however, the principles of care apply broadly to any medications in the opioid class. The “perioperative time frame” refers to administration of opioids as part of the anesthetic for an operation and the recovery period. The length of time will be different based on variations in the surgeries involved and multiple patient factors including age and pain sensitization. For the purposes of this paper, the recovery time frame will be defined as the period for which most surgical patients would be expected to require opiates for each specific procedure. For major surgeries, this would include time as an inpatient with emphasis on parenteral administration of opioids during that time period. We will also address the brief time pediatric postoperative patients receive oral opioids at home. We will address the treatment of chronic pain patients that come for surgery but not the use of opioids for, or the treatment of, chronic pain that might develop after surgery.

## 3 focus

These recommendations are intended to address questions related to the use of opioid medications for children undergoing surgery or painful procedures. This document is not comprehensive in nature. We have chosen to highlight the areas of appropriate opioid delivery methods, monitoring, and pain assessment since these aspects have high variability between care delivery systems and bear most directly on the safe use of these agents. Issues involving children with chronic pain and pain sensitization as well as previous opioid therapy are also addressed since these patients are often the most difficult to manage in the perioperative time frame.

## 4 purpose

These recommendations are intended to enable maximum patient benefit from opioid medications in order to provide potent analgesia and best outcomes for children after surgery.^11^, ^12^ We also intend to provide appropriate caution concerning the potential serious side effects associated with their administration.^7^ The collective purpose is to promote appropriate and safe practice, while also dispelling unreasonable fear of their administration.

## 5 task force members and consultants

The SPA board approved the appointment of nine primary authors to review the published evidence and make recommendations on issues related to the safe use of opioids in children. Those appointed come from a diverse array of institutions and sub‐specialty backgrounds including pain medicine, clinical pharmacology, clinical trials, addiction medicine, general operating room anesthesia, and perioperative nursing. We also engaged four senior anesthesiologists with extensive histories of contribution to the specialty of pediatric anesthesiology, pain management, and opioid investigation.

## 6 topics and strength of evidence

The expert panel was convened and met in person twice prior to beginning the process of formulating these recommendations. The members agreed that this manuscript should focus on the *perioperative* time frame and specifically on the process of opioid delivery and monitoring of patients treated with opioids. The panel also agreed the document should attempt to target those issues most pressing for health care providers who manage pediatric patients in the perioperative time frame. Selected topics were to include age‐related effects of opioids; patient‐controlled analgesia (PCA); postoperative outpatient opioid administration; monitoring of patients on opioids; pain assessment and opioid efficacy; opioid side effects and treatment; perioperative management of patients on chronic opioids; and management of patients with opioid addiction. Several sections that were initially written for this manuscript were eliminated due to editorial considerations of overlapping content with other published reports.

The members of the taskforce searched academic databases for relevant articles on opioid administration in children associated with surgical pain relief including effectiveness, techniques for delivery, monitoring, and side effects. A list of the databases searched and the search terms used is presented in Table A1. Age limits of 0‐18 years were set however, adult data were included when the collected opinion of the taskforce determined that pediatric evidence base was lacking, but adult studies offered robust, reliable data. Recommendations were downgraded when evidence was from adult cohorts. All papers present in the databases were eligible for inclusion with no “beginning” date and ending January of 2016. Each study was classified as retrospective, prospective observational, prospective randomized, or prospective, randomized and blinded. One author was responsible for the literature search for each section and reviewed the papers that were generated. This individual then determined which papers provided evidence that was appropriate for the section. Many older papers were judged to involve medications or modalities that were not relevant to the current standard of care. The written text for each section (along with the references to support the text) were evaluated by all members of the taskforce including consultants. The group specifically reviewed the type of studies that were available to support the recommendations in each section and considered the level of evidence as proposed by the author. The taskforce met in person four times and there were six conference calls arranged to facilitate these discussions. Where there was a question as to whether a study should be included, it was discussed and negotiated until all members felt comfortable with the decision to include or reject. Similarly, text for each section including the recommendations were adjusted until there was unanimous agreement among the members of the committee and the consultants. Recommendations that could not be unanimously agreed upon were not included. After the document was completed, the entire manuscript was reviewed by the Board of Directors of SPA. Board members offered editing recommendations and feedback, but did not fundamentally change the level of evidence or the nature of the recommendations.

The strength of evidence available for specific recommendations was based on a three‐tiered classification system that we patterned after evidence‐based practice guidelines from the American Society or Anesthesiologists^13^:

- Level A: Based on prospective, randomized trials, or pharmacokinetic and pharmacodynamic trials that meet the highest standards for investigation and reporting.
- Level B: Based on prospective, observational data or appropriately conducted retrospective analysis with consideration of appropriate confounders and compensation for the influence of these factors.
- Level C: Based on case reports, case series, or consensus involving experts who presented a well‐defined process for formulating conclusions.

For each recommendation, we have taken the level at which the preponderance of evidence was derived and clearly designated it as such. A list of the recommendations and strength of evidence is provided in Table 1.

**Table 1:** Society for Pediatric Anesthesia Opioid recommendations for the use of opioids in children during the perioperative period with strength of evidence noted

Recommendation	Level of evidence
Age related pharmacokinetic and pharmacodynamic opioid effects	
A validated, age‐adjusted morphine dosing regimen should be used for all pediatric patients but particularly for neonates where the dose and dosing interval will need to be altered significantly.	A
Dosage of most synthetic opioids should be decreased in neonates during the first two to four weeks of life (and for premature neonates until at least 44 weeks post conceptual age). For remifentanil, the effective half‐life in neonates is similar to that of older children and adults, and thus requires no adjustment. There is sparse information on methadone, but it appears to have similar pharmacokinetics across all age ranges.	B
Dosage of most synthetic opioids should be decreased in neonates during the first two to four weeks of life (and for premature neonates until at least 44 weeks post conceptual age). For remifentanil, the effective half‐life in neonates is similar to that of older children and adults, and thus requires no adjustment. There is sparse information on methadone, but it appears to have similar pharmacokinetics across all age ranges.	B
The use of patient controlled analgesia (PCA)	
The use of PCA opioid delivery is preferable to IM opioid delivery for perioperative pain control. IM administration of opioids is not recommended as a primary pain control modality.	B
PCA opioid delivery is safe, efficacious, and correlated with higher patient satisfaction when compared to intermittent intravenous opioid analgesia.	B
There is insufficient and conflicting evidence to recommend the use of a specific opioid over another for PCA post‐operative pain control. Due to the risk of accumulation of toxic metabolites (normeperidine) that may cause seizures, meperidine is not recommended by our expert panel.	B
There is conflicting and insufficient evidence to indicate a difference in overall analgesia, sleep patterns, or adverse events with the addition of continuous opioid infusion to PCA in children. Use of a basal infusion should be individualized based on consideration of the clinical situation, pain severity, and risk factors.	B
There is evidence that nurse controlled analgesia and parent controlled analgesia is associated with safety and efficacy outcomes that are similar to that of standard PCA therapy. These methods must be applied in an institutionally‐sanctioned program with appropriate training and monitoring.	B
The use of ketorolac should be strongly considered as an adjunct to PCA for pediatric perioperative pain control. Most evidence available for NSAID effect on PCA dosing involves ketorolac, however there is good reason to assume another NSAID would have a similar PCA dose sparing effect.	A
The use of acetaminophen should be considered as an adjunct to PCA for pediatric perioperative pain control.	A
Recommendations for outpatient post‐operative opioid use in children	
Educational resources must be provided to inform parents of the appropriate indications for pain medications and strategies for the safe use of opioids, non‐opioids and other measures to manage their child’s post‐operative pain. Parents should receive both verbal and written detailed discharge instructions regarding home pain management with instructions regarding safe storage and disposal of leftover medications.	C
Here is evidence to advise against the use of tramadol for specific populations of pediatric patients, particularly young patients (under 12) and those with OSA.	B
There is insufficient evidence to recommend PRN vs. scheduled dosing strategies for opioids after surgery in children. Expert consensus is to use a PRN strategy until further evidence is available.	C
Opioid pain medications should not be prescribed with benzodiazepines except in children for whom there is a specific indication and alternative treatment options are inadequate. Doses should be limited to the lowest effective level and parents should be warned about the potential for excessive sedation and respiratory depression.	C
Opioid prescriptions should be limited to that required for the expected period of severe pain after surgery (local laws may set limits for quantity or time parameters for opioid prescriptions). Patients should be educated concerning the appropriate storage and disposal of opioids.	B
Opioid treatment of the patient with chronic pain scheduled for major surgery	
For pediatric patients with chronic pain who are maintained on opioids, continue established preoperative dosing during the perioperative period as a baseline. Acute post‐surgical analgesia should be provided over and above the baseline opioids. Use of non‐opioid analgesia is encouraged including regional analgesia techniques, alpha‐2 agonists, ketamine, acetaminophen, nonsteroidal anti‐inflammatory drugs, and neuropathic pain medications such as gabapentinoids or antidepressants.	C
If a patient with chronic pain on opioid therapy undergoes surgery and the patient’s underlying pain source was independent of the surgery, the patient’s baseline pain should continue to be managed by the physician who had been doing so preoperatively. Opioid analgesics for the perioperative pain, if needed, should be prescribed in limited quantities consistent with the degree of physiologic trespass.	C
For pain management of patients with central sensitization – strategies should be similar to those for pediatric pain patients on chronic opioids (use non‐opioid analgesic techniques to the greatest extent possible). Opioids should be prescribed as needed. These patients benefit from the involvement of a pain physician for the purposes of assuring appropriate use and discontinuation of medications in a reasonable time frame.	C
Assessment of pain and analgesic efficacy	
Regular pain assessments should be part of the perioperative care/treatment of pediatric patients who are receiving opioid medications. These assessments should be made using validated measures. The pain assessment should consider the unique circumstances of the child’s psychological state and the extent of surgery.	B
Pain intensity scores can be used to assess a child’s perceived degree of discomfort. However, decisions to administer analgesics should take into consideration patient functional behaviors, situational factors such as pain source, self‐reported pain scores, parental observation, and other potential sources of distress, rather than arbitrarily selected pain score cutoffs.	B
Behavioral observation can be used to assess pain‐related distress in children. Directions to administer analgesics in children who cannot self‐report should take into consideration situational factors such as pain source, observational pain scores, and other potential sources of distress, rather than arbitrarily selected pain score cut‐offs.	B
Changes in pain scores should be used in conjunction with other verbal/behavioral measures as indicators of pain relief and analgesic response (e.g. side effects) when making analgesic decisions.	B
Physiologic parameters should be used to assess the child’s nociceptive response when it is not possible to assess pain with self‐report of behavioral measures (e.g., when the child is sedated or is receiving neuromuscular blockers). It is recommended that other potential sources of physiologic distress (e.g., emotional distress, hypovolemia, fever, hypercarbia) be considered and/or ruled out when making treatment decisions.	B
A child’s functional recovery should be assessed to inform treatment plans.	C
Assessing pain location is recommended to differentiate incisional pain from other potential sources of postoperative pain. The nature of pain should be assessed to assist in the differentiation of pain type.	B
Monitoring of patients on perioperative opioid therapy	
Physiologic monitoring of children receiving initial intravenous opioid treatment should include pulse oximetry for the first 24 hours. Continuous monitoring of respiratory rate and ECG should be considered in pediatric patients who are on oxygen, or who have risk factors for respiratory depression. Physiological monitoring may be suspended when the patient is alert, awake, and/or ambulating.	C
Although not specifically addressed in the literature, the expert panel recommends frequent assessment of the quality, as well as the rate of respirations, should be performed by direct observation and recorded in the medical record. Increased frequency and intensity of these observations is recommended for children in high‐risk groups in addition to standard electronic monitoring.	C
Patients with obstructive sleep apnea, obesity (>95 percentile BMI), and recurrent nighttime oxygen desaturations are at higher risk for opioid induced respiratory depression. Opioid dosing should be based on ideal or lean body weight and the dose of opioid should be reduced by 50% to 67%. Additionally, extended respiratory monitoring is required when opioids are being administered to this population in the perioperative period.	B
Patients receiving opioid analgesia perioperatively should have regular assessment of their level of sedation using a validated sedation score that evaluates level of alertness or mentation, rather than utilizing a procedural sedation scale. This rating should be part of the medical record along with measures of pain and physiological status.	C
Review of code team calls or emergency response calls and delivery of emergent naloxone doses should be part of institutional efforts to critically evaluate and reduce preventable opioid‐related adverse events.	B
Opioid side effects in children	
Naloxone infusion is helpful in treating and possibly preventing opioid‐induced pruritus.	A
Expert consensus supports the use of nalbuphine although there is conflicting evidence concerning its effectiveness at this time.	C
Opioid use should be minimized where possible to decrease the incidence of nausea and vomiting.	A
Naloxone infusion should be considered for intravenous opioid therapy for the prevention or treatment of nausea and vomiting.	A
It is reasonable to consider common anti‐emetic medications for the treatment or prevention of nausea and vomiting while on intravenous opioid therapy. Preference should be for non‐sedating medications.	A
Postoperative opioid‐induced ileus may be improved with methylnaltrexone in infants and children.	B

## 7 age‐related pharmacokinetic and pharmacodynamic opioid effects

To be discussed in this section:

- What is the evidence for changes in opioid pharmacokinetics and pharmacodynamics with age?
- How should opioid dosing be adjusted with age?
- Specifically, what is the effect of age on the issue of opioid induced respiratory depression?

Recommendations for dosing opioids in children are cited in a number of publications.^6^, ^14^, ^15^ The appropriate dose for a given patient will vary with many factors, including the invasiveness of the surgery, adjunctive medications, previous exposure to opioids, and underlying illnesses.^7^, ^16^ The pharmacokinetics and pharmacodynamics of opioid agents also varies with age. Multiple investigators have evaluated age‐related opioid pharmacokinetics and pharmacodynamics primarily using observational methodologies.^17^, ^18^ It is important to note that among these studies there is a poor correlation between measured blood opioid concentrations and patient analgesia. Many factors confound these studies and contribute to the lack of correlation. Most prominent among these is that pain perception is subjective (anxiety and constitutional pain sensitivity play a role) and the tools used to study pain in children vary widely. In spite of this, there are some consistent findings with respect to opioid pharmacokinetics and pharmacodynamics that are important to consider when providing perioperative pain control with these medications.

### 7.1 Pharmacokinetics/pharmacodynamics of morphine in children

Morphine is a commonly employed agent for pain control in newborns, infants, and children. There is consistent evidence that the greatest variation in pharmacokinetic parameters occurs in neonates and very young infants. Neonates have a larger volume of distribution and decreased protein binding which results in a greater free fraction of the opioid. Glomerular filtration rate (GFR) and hepatic enzyme development are relatively immature in neonates (0‐28 days of life) resulting in a decreased clearance of morphine at approximately one‐fifth to less than one‐tenth of that in older children and adults.^19^, ^20^ There is considerable evidence from multiple observational trials that indicating morphine clearance is increased via glucuronidation in older children when compared to neonates.^14^ Notably neonates have consistently been shown to have at least a 50% lower glucuronidation rate, which contributes to a lower clearance. In one study, following a single intravenous dose of morphine (0.1 mg/kg) to 20 neonates with a gestational age of 26‐40 weeks, the free fraction of drug was 80% compared to 65% in adults. This finding, combined with lower morphine clearance, mandates longer dosing intervals in this age group.^21^, ^22^ In addition, the primary metabolite of morphine (morphine‐6‐glucoronide [M‐G‐6]) has a greater analgesic and respiratory depressant effect than morphine. These effects are amplified in the setting of decreased renal clearance typical of neonates.^23^ Finally, opioid dosing should take into consideration that the blood brain barrier is more permeable in neonates, leading to the potential for less fat soluble opioids to cause increased risk of respiratory depression. Using a nonlinear dosing regimen, a relatively predictable serum level of morphine can be achieved in pediatric patients in spite of a broad range of clearance rates.^24^ An evidence‐based model has been validated using multiple large datasets involving pediatric patients who received morphine for pain control.^25^ A dosing regimen based on this model has been prospectively validated.^26^ In this regimen, doses for neonates under 10 days of life receive 50%‐70% lower doses compared to older patients. Efficacy has been shown to be maintained with this regimen while risk of overdosing is decreased. Neonates with increased abdominal pressure or use of vasoactive medications may further delay clearance due to changes in hepatic blood flow.^17^ Maturation of clearance and underlying metabolic pathways occurs relatively rapidly during infancy and reaches the levels of older children by approximately 6 months of age.^17^, ^19^, ^23^ Children 2‐11 years of age actually have a higher clearance rate and larger volume of distribution than older children or adults.^27^

*Recommendation*: A validated, age‐adjusted morphine dosing regimen should be used for all pediatric patients but particularly for neonates where the dose and dosing interval will need to be altered significantly.

*Strength of evidence*: A.

### 7.2 Pharmacokinetics/pharmacodynamics of opioids other than morphine

There is considerably less evidence concerning pharmacokinetics and pharmacodynamics of opioids other than morphine. Data on fentanyl, sufentanil, and alfentanil indicate each of these drugs undergo much more rapid clearance in infants and older children relative to neonates.^28^, ^29^ Additionally, there is evidence that premature and term neonates have considerably lower clearance of alfentanil when compared to infants and children.^30^ On the other hand, remifentanil clearance is greatest in neonates and decreases with age while volume of distribution has an opposite trend yielding half‐lives that appear similar across all age groups.^31^ There are no data on the safety or effectiveness of synthetic opioids in pediatric patients, other than fentanyl, when used outside of the perioperative time frame. Their use can not be recommended in other clinical situations.

The pharmacokinetics of methadone has been studied in adolescents undergoing major spinal surgery and appears to be similar to that in adults including an elimination half‐life of 44.4 hours.^32^, ^33^ There are sparse data on the pharmacokinetics or pharmacodynamics of methadone vs other (shorter acting) opioids for perioperative pain control in children. A small study of postoperative patients demonstrated a propensity for more prolonged respiratory depression after methadone when compared to pethidine or morphine.^34^ One prospective, randomized, blinded study comparing methadone to morphine administered in the PACU found that patients who received methadone perioperatively had lower pain scores and required fewer supplemental medications in the 36 hours following their PACU stay.^35^ In this study of 35 patients, there were no major adverse events. Another investigation evaluated the accumulated information on methadone pharmacokinetics in infants and children found no evidence of maturation of clearance with age.^36^

*Recommendation*: Dosage of most synthetic opioids should be decreased in neonates during the first 2‐4 weeks of life (and for premature neonates until at least 44 weeks post conceptual age). For remifentanil, the effective half‐life in neonates is similar to that of older children and adults, and thus requires no adjustment. Methadone pharmacodynamics effects are prolonged compared to morphine and the pharmacokinetic profile appears to be consistent across pediatric age ranges.

*Strength of evidence*: B.

### 7.3 Respiratory depression

Evidence concerning the respiratory depressant effects of opioids in children vs adults is sparse. In one study, Lynn et al evaluated the respiratory depressant effects in 30 newborns‐toddlers receiving morphine infusions after cardiac surgery. No age‐related differences in respiratory effects were observed at similar morphine concentrations.^37^ Similarly, in a small study of pediatric patients (11 days to 7 years of age) receiving morphine after major surgery, Olkkola et al found no difference in the respiratory depressant effects of morphine between the youngest and oldest patients.^19^ In a study of fentanyl‐related respiratory depression, Hertzka et al found the elevation of PaCO_2_ correlated with increasing plasma fentanyl concentrations, but did not differ between groups of infants vs children (1‐5 years of age) vs adults.^38^ The authors concluded there is no evidence of increased sensitivity to respiratory depression in children over 3 months of age when compared to adults. On the other hand, a case review of pediatric patients with opioid‐related respiratory depression found that age less than 1 year is an independent risk factor. Other similar reports lacked serum drug level data.^39^

*Recommendation*: Term infants (older than 3 months of age) are not at increased risk of respiratory depression due to opioids when compared to older children and adults *at the same blood concentration of opioid*. The dose of opioids (dose/kg) should be similar to older infants and children after 6 months of age (considering underlying health and previous exposure to opioids). When beginning opioid dosing in infants younger than 3 months of age, the patient should be in a highly monitored environment (such as a step‐down unit, PACU, or ICU/NICU).

*Strength of evidence*: B.

## 8 the use of pca

Issues to be discussed in this section:

- How does PCA opioid delivery compare to other modes of opioid delivery?
- What is the best opioid to use for PCA therapy?
- What is the evidence surrounding the use of NCA or parent‐controlled analgesia?
- What kinds of adjunctive medications should be added to PCA opioid therapy?

### 8.1 Introduction—PCA vs intramuscular opioid delivery

Patient‐controlled analgesia delivery of opioids has become the standard for analgesia after major surgery in children. This section will compare this delivery method vs other opioid delivery modalities. There are many adult studies comparing PCA to intramuscular (IM) opioids. One meta‐analysis examined 15 randomized controlled trials including 787 adult patients found PCA provided greater patient satisfaction and improved pain relief, as reported by visual analog scale (VAS) scores, and no difference in side effects.^36^ Three pediatric studies were identified but were not randomized or controlled.^40^, ^41^, ^42^ These studies found improved pain control with PCA, as measured by VAS, despite similar consumption of opioid equivalents. None of these studies found a higher level of adverse events for PCA use as compared to IM opioids. One study found a higher level of sedation in the IM morphine group compared to PCA.^40^

*Recommendation*: The use of PCA opioid delivery is preferable to IM opioid delivery for perioperative pain control. IM administration of opioids is not recommended as a primary pain control modality.

Strength of Evidence: B.

### 8.2 PCA vs intermittent IV opioids

There were no randomized controlled studies identified that compared PCA with intermittent opioid administration in the pediatric population. A number of cohort studies supported the use of PCA as efficacious and with acceptable rates of adverse events in a variety of postoperative settings (abdominal, laparoscopic, thoracic, orthopedic surgery) and with a variety of opioid choices (meperidine, fentanyl, morphine).^43^, ^44^, ^45^, ^46^, ^47^, ^48^, ^49^, ^50^, ^51^, ^52^, ^53^, ^54^, ^55^, ^56^ Higher quality data exist for adults. Meta‐analysis of randomized controlled trials in the adult perioperative population, performed for the purpose of updating the American Society of Anesthesiologist's (ASA) guidelines, found no analgesic benefit of PCA over nurse‐administered IV opioids.^57^ A Cochrane review of 49 studies and 3412 patients comparing PCA to intermittent dosing for postoperative pain showed that patients who received PCA reported lower pain scores (nine to 10 points lower on a 100‐point VAS), greater satisfaction, but used more morphine equivalents (7 mg more in the first 24 hours). There was no difference in adverse events except for a higher incidence of pruritus (15% vs 8%).^58^, ^59^, ^60^ Finally, Faerber et al used propensity matched cohorts to evaluate major adverse events and found that PCA was associated with a lower risk of cardiopulmonary resuscitation (CPR) or the need for mechanical ventilation when compared to intermittent IV dosing.^59^

*Recommendation*: PCA opioid delivery is safe, efficacious, and correlated with higher patient satisfaction when compared to intermittent intravenous opioid analgesia.

*Strength of evidence*: B (largely based on adult data).

### 8.3 Opioid selection for PCA in children

The evidence is sparse and inconclusive concerning the choice of opioid for PCA in children. One randomized controlled trial of morphine vs meperidine in 50 orthopedic patients 8‐16 years old showed no difference in side effects, but lower numerical rating scale pain scores in patients receiving morphine.^60^ Another randomized controlled study of 60 children posttonsillectomy compared tramadol to morphine, with poorer pain control, similar sedation levels, and lower nausea scores in the tramadol cohort.^61^ Comparison of morphine and hydromorphone in a randomized controlled trial of 96 pediatric patients showed no difference in pain control, as reported by VAS, with morphine equivalents administered (assuming 1:5 potency ratio), or side effects (hypoxia, pruritus, nausea, urinary retention).^62^ A retrospective comparison study of morphine and hydromorphone including 514 pediatric patients at a single center revealed more frequent conversion from morphine to hydromorphone due to pruritus and poor pain control.^63^ The nature of this study did not allow for a determination of overall superiority of one drug vs another in terms of pain control or side effects.

*Recommendation*: There is insufficient and conflicting evidence to recommend the use of a specific opioid over another for PCA postoperative pain control. Due to the risk of accumulation of toxic metabolites (normeperidine) that may cause seizures, meperidine is not recommended by our expert panel.^64^

*Note*: Meperidine use for postoperative shivering is not addressed in this recommendation.

*Strength of evidence*: B.

### 8.4 Use of continuous infusion simultaneous with PCA

Randomized controlled trials that have compared continuous opioid infusion with PCA to PCA alone have suggested that the addition of continuous infusion may have many possible effects on the overall impact of PCA therapy. Comparative studies of 6‐12 year olds postappendectomy^65^, ^66^; 5‐20 year olds postelective spinal fusion for scoliosis^67^; school‐age children postlower extremity orthopedic surgery^68^; and 6‐15 year olds postappendectomy^69^ fail to show an overall improvement in pain scores with the addition of a continuous infusion. There is evidence of an increase in overall opioid usage and an increased level of sedation with continuous infusions. The impact on sleep has been inconsistent. Some data indicate an increase in sleep duration^65^, ^66^ but there has also been documentation of an interruption of normal sleep patterns in a single‐center retrospective audit of 126 children.^70^

In terms of the risk of adding continuous infusion to PCA, a meta‐analysis of randomized controlled trials (including both adult and pediatric patients) indicated that pediatric patients have an overall lower risk of serious adverse events associated with a continuous infusion combined with PCA when compared to the adult population.^71^ This finding may be confounded by routine use of continuous respiratory monitoring in the pediatric population.^72^ Another meta‐analysis examining randomized controlled studies comparing PCA vs PCA plus background infusion for postoperative children showed no difference in pain scores, opioid consumption, or risk of adverse events at 12 and 24 hours.^73^

Recommendation: There is conflicting and insufficient evidence to indicate a difference in overall analgesia, sleep patterns, or adverse events with the addition of continuous opioid infusion to PCA in children. Use of a basal infusion should be individualized based on consideration of the clinical situation, pain severity, and risk factors.

Strength of Evidence: B.

### 8.5 PCA‐by nurse or parent proxy—(Nurse‐controlled analgesia and parent‐controlled analgesia)

Many pediatric patients are either developmentally or physically unable to activate their PCA pump and require assistance from a caregiver. When control of the bolus dose delivery is turned over to a caregiver, the technique is referred to as PCA by proxy (PCA‐P) or NCA. One retrospective study of 302 children managed with NCA vs intermittent IV dosing documented similar numbers of adverse events in both cohorts. This study was confounded by the fact that the NCA cohort tended to be younger and had more medical co‐morbidities.^74^ A prospective 1‐year observational study of 212 children undergoing nurse or parent‐controlled analgesia showed these methods were effective for pain control. Notably, all participants underwent extensive standardized training in the use of PCA‐P. Minor adverse events occurred in 4% of patients.^75^ A large retrospective review of 10 079 children (average age 4 years) and including 510 neonates, revealed 39 serious adverse events that required resuscitation and naloxone administration. Of these 13 were infants less than 3 months old and four were ex‐premature neonates and no deaths occurred.^76^ Another study of 107 preschoolers and infants utilizing nurse or parent‐controlled analgesia found 1.9% of those patients had a serious adverse event requiring naloxone administration.^77^ NCA has also been investigated in the neonatal intensive care unit (NICU) in a single retrospective study of 20 infants compared to 13 who had fentanyl continuous infusions. The infants receiving NCA had excellent pain control and a significantly lower opioid consumption than those on continuous infusion (84% less morphine equivalents). There was no difference in occurrence of adverse events or in subsequent methadone use.^78^ One randomized controlled trial of PCA vs PCA with continuous infusion in children after spinal fusion included a subgroup analysis that indicated nurses who performed NCA underestimated patient pain as compared to self‐reported pain scores and administered less total opioid than patients self‐administering medication.^67^

The use of authorized *parent‐controlled* analgesia has been associated with acceptable safety and effective postoperative analgesia delivery for children who are unable to use PCA themselves.^67^, ^74^, ^75^, ^76^, ^77^, ^78^, ^79^, ^80^, ^81^, ^82^, ^83^, ^84^ A dose‐finding study of 30 infants and toddlers (6 months to 2 years of age) using parent‐controlled analgesia with fentanyl after cleft palate repair had no instances of over‐sedation or apnea and three patients with vomiting.^82^

*Recommendation*: There is evidence that NCA and parent‐controlled analgesia is associated with safety and efficacy outcomes that are similar to that of standard PCA therapy. These methods must be applied in an institutionally sanctioned program with appropriate training and monitoring.

*Strength of evidence*: B.

### 8.6 PCA adjunctive medications

#### 8.6.1 Ketorolac

There is supportive evidence for the opioid sparing effect of ketorolac when added to PCA analgesia. A randomized controlled study of 68 children comparing ketorolac to placebo every 6 hours found better pain control and a 42% reduction in morphine administered by PCA after PACU discharge.^85^ Another study of 50 pediatric patients found that a single dose of ketorolac intraoperatively was superior to placebo resulting in lower pain scores and decreased PCA morphine requirements in the first 12 hours.^86^ A case‐control study comparing 29 children who received ketorolac plus morphine to children who received only morphine similarly found an opioid sparing effect of ketorolac without a difference in side effects.^87^ A randomized controlled trial of 35 adolescents after spinal fusion found lower postoperative pain scores and decreased morphine consumption delivered by PCA when ketorolac was included in their therapy.^88^ There was no evidence for excessive bleeding with ketorolac in any of these studies.

*Recommendation*: The use of ketorolac should be strongly considered as an adjunct to PCA for pediatric perioperative pain control. Most evidence available for nonsterioidal anti‐inflammatory drug (NSAID) effect on PCA dosing involves ketorolac, however there is good reason to assume another NSAID would have a similar PCA dose sparing effect.

*Strength of evidence*: A.

#### 8.6.2 Acetaminophen

There is supportive evidence for an opioid sparing effect when acetaminophen is added to PCA therapy in pediatric patients. A study of 80 children after appendectomy showed a significant morphine sparing effect with diclofenac, but a nonsignificant effect (17% less) with oral acetaminophen.^89^ In contrast, a study of 63 children after ureteroneocystostomy, compared fentanyl with intravenous acetaminophen PCA to fentanyl‐only PCA found that pain scores were similar between the groups. However, a fentanyl sparing effect (53% reduction) was observed along with a lower incidence of vomiting and sedation in the fentanyl‐acetaminophen group.^90^ In another study of 36 children and adolescents after spinal fusion surgery, those receiving three postoperative doses of intravenous acetaminophen were compared to placebo. Children who received acetaminophen showed a reduction in oxycodone consumption as administered by PCA and improved pain scores.^91^

There is evidence that acetaminophen is less effective for neonates than older children, particularly for procedural pain.^92^, ^93^

*Recommendation*: The use of acetaminophen should be considered as an adjunct to PCA for pediatric perioperative pain control.

*Strength of evidence*: A.

## 9 recommendations for outpatient postoperative opioid use in children

Topics in this section:

- What is the role of educational resources?
- What is the evidence for the choice of specific opioid therapy, dosing strategies for outpatient opioids, use of combined opioid and benzodiazepine therapy, or the prescribing practices for opioid medications for outpatients?

### 9.1 Educational resources

There is evidence that pain is poorly managed at home after surgery in children.^94^ Data from multiple investigators indicate children have been shown to report significant pain after many types of surgeries including tonsillectomy or adenotonsillectomy (T&A), dental extraction, and circumcision. Studies suggest that parents may not give adequate pain medications to children for a number of reasons, including lack of information, inadequate understanding of perioperative opioid drug requirements, inadequate or inappropriate understanding of dosing, uncertainty over adverse drug effects, and child refusal.^94^, ^95^, ^96^ Poor understanding of opioids can lead some parents to withhold opioids altogether to avoid perceived risk or, conversely, to administer opioids when children are excessively sedated.^97^, ^98^

Educational materials can take many forms. In adult patients the use of an informational brochure has been shown to significantly improve the percentage of patients who disposed of unused opioid pain medications after surgery.^99^ For children, websites such as kidshealth.org, provide simple, easy to read answers to the most common questions parents have about opioids for pain treatment. Additional links are included to provide additional information as needed. More recently websites, such as http://mychildisinpain.org.uk, combine excellent graphics with informative text and video presentations to provide up to date information on how parents should approach pain management and include specific information on medication issues.

*Recommendation*: Educational resources must be provided to inform parents of the appropriate indications for pain medications and strategies for the safe use of opioids, nonopioids and other measures to manage their child's postoperative pain. Parents should receive both verbal and written detailed discharge instructions regarding home pain management with instructions regarding safe storage and disposal of leftover medications.

*Strength of evidence*: C.

### 9.2 Specific opioid therapy for outpatients

There is a paucity of pediatric literature upon which to formulate evidence‐based recommendations for opioid dosing after surgery and discharge home. One exception is the strong evidence against the use of codeine, particularly in children undergoing tonsillectomy and for other surgeries.^100^, ^101^, ^102^ Metabolism is variable with 1% of ethnic Northern Europeans and up to 29% of Ethiopians, experiencing ultra‐rapid metabolism of codeine to morphine. This results in high morphine levels even after standard codeine doses. On the other hand, approximately 10% of patients lack the ability to metabolize codeine to morphine and therefore experience little analgesic benefit from this drug.

Similarly, tramadol is a prodrug metabolized by enzyme complexes CYP3A4 and predominantly 2D6. Like codeine, there can be ultra‐rapid metabolizers for CYP2D6 leading to apnea. It also carries a risk of seizures.^103^, ^104^ There is a common misunderstanding that it is a safer alternative to opioids.^7^ The FDA has issued a warning concerning the use of tramadol that advises restricted use in children younger than 18 years after tonsillectomy, children younger than 12 years of age, and adolescents between 12 and 18 years who are obese, have obstructive sleep apnea (OSA), or lung disease.^105^, ^106^

*Recommendation*: Codeine should be avoided in children and nursing mothers as a postoperative analgesic. This is particularly true if they have symptoms of obstructive sleep apnea or sleep disordered breathing.

*Strength of evidence*: A.

*Recommendation*: There is evidence to advise against the use of tramadol for specific populations of pediatric patients, particularly young patients (under 12) and those with OSA.

*Strength of evidence*: B.

### 9.3 Dosing strategies

There are only two randomized prospective studies evaluating the appropriate dosing of opioids in postoperative pediatric patients. In one study, Sutters et al compared “around the clock” (ATC) dosing to an “as needed” administration (PRN) strategy. In this case, children who received ATC acetaminophen with hydrocodone were compared to those who received the same medications “as needed” for complaints of pain.^107^ There was improved pain control in the ATC cohort, but a slightly higher incidence of complications including a higher rate of daytime sedation in this group. A Cochrane review of ATC vs “as needed” dosing,^108^ including three RCTs with a total of 246 children aged under 16 years of age found more medication is administered in the ATC group, but were unclear as to whether or not pain management was improved.

*Recommendation*: There is insufficient evidence to recommend PRN vs scheduled dosing strategies for opioids after surgery in children. Expert consensus is to use a PRN strategy until further evidence is available.

*Strength of evidence*: C.

### 9.4 Combinations of opioids and benzodiazepines

A recent FDA review determined that the use of opioid medications in combination with benzodiazepines in adults has increased, and that this practice has resulted in serious side effects.^109^ Two studies are highlighted in this statement. The first found that the number of opioid analgesic prescriptions increased 8% and the annual number of benzodiazepine prescriptions increased by 31% over the last 5 years.^110^ The number of patients receiving overlapping prescriptions for these two medications has increased by 41%. The second study found that between 2004 and 2011 the rate of opioid overdose deaths, in which benzodiazepines were also implicated, increased from 18% to 31%.^111^ However, no similar pediatric data have been reported.

*Recommendation*: Opioid pain medications should not be prescribed with benzodiazepines except in children for whom there is a specific indication and alternative treatment options are inadequate. Doses should be limited to the lowest effective level and parents should be warned about the potential for excessive sedation and respiratory depression.

*Strength of evidence*: C.

### 9.5 Opioid prescribing

Several federal agencies and physician organizations (Centers for Disease Control [CDC], Substance Abuse and Mental Health Services Administration [SAMHSA], American Medical Association [AMA]) have weighed in on the over prescription of opioids for postoperative patients. These organizations highlight the growing incidence of opioid overdose in pediatric patients, particularly teenagers. The most common source of prescription opioids is drug supplies from leftover prescriptions (family, friends, neighbors, etc) where opioid prescriptions were overly generous in order to avoid any possible requirement for refilling prescriptions. Recent studies have found children are prescribed amounts of opioids that significantly exceed the total administered at home. Parents rarely received instructions regarding safe disposal of leftover opioids.^112^

*Recommendation*: Opioid prescriptions should be limited to that required for the expected period of severe pain after surgery (local laws may set limits for quantity or time parameters for opioid prescriptions). Patients should be educated concerning the appropriate storage and disposal of opioids.

*Strength of evidence*: B.

## 10 opioid treatment of the patient with chronic pain scheduled for major surgery

Topics in this section:

- What are the strategies for providing analgesia for patients with chronic pain?
- How do we approach analgesia for patients with central sensitization?

Many pediatric patients present for surgery with a history of chronic pain, be it pain related to “central sensitization”,^113^ cancer pain,^114^ chronic orthopedic conditions or other medical issues.^115^ Some of these children will be opioid dependent or tolerant due to their treatment with these medications on a long‐term basis. Furthermore, pediatric patients with chronic pain who are on long acting or partial agonist opioid therapy, including methadone or buprenorphine, require special consideration.^116^ There is little evidence to guide the treatment of these patients, but experience and reason demands a flexible and multimodal approach.^117^

It is critical to differentiate certain terms associated with chronic opioid use. Physical dependence describes the alterations in physiologic response that result from opioid binding and receptor‐mediated activity. In this subgroup, sudden discontinuation of opioids leads to opioid withdrawal or abstinence syndrome. Opioid tolerance refers to the pharmacologic adaptation that occurs after chronic exposure to opioids where there is a shift in the dose‐response curve and patients require increasing amounts of drug to maintain the same pharmacologic effects. Finally, drug addiction refers to a complex phenomenon where the use of a drug becomes the central point of focus for the user's life (ie, deriving psychological reward other than analgesia) even in the face of obvious physical or psychological harm. Patients who exhibit addiction are usually physically dependent on an opioid. Dependence and addiction may exist in conjunction with each other or as independent entities and must be identified and appreciated in order to appropriately (and safely) treat pain in this challenging population.^118^

### 10.1 Perioperative care of patients with chronic pain disorders who are taking opioids

Recommendations for pediatric patients presenting for surgery who are on opioids for chronic pain syndromes are entirely extrapolated from the adult literature. There is a single observational study showing that pediatric patients previously placed on opioid infusions in an intensive care unit do not have increased perioperative opioid requirements if they have previously been successfully weaned off the opioids.^119^

In the adult literature, recommendations for perioperative management of patients with chronic pain using opioids are based on case reports, common sense, or consensus rather than randomized studies. Although not scientifically tested, these recommendations have been advocated in the setting of receptor down‐regulation and appreciation of the physiological and psychological changes that accompany chronic pain and opioid treatment. In all cases, perioperative care is advised to begin with preoperative administration and continuation of the daily maintenance (or baseline) opioid dose.^120^ Additional doses of opioid should be titrated intra‐operatively and postoperatively to provide effective postsurgical analgesia in addition to covering the baseline requirements.^118^ Dosing guidelines are not available, but requirements to meet postsurgical analgesic requirements are affected by receptor down‐regulation and may need to be increased three to 10 times of that required by opioid naïve patients.^121^, ^122^ Continuous opioid infusion or patient‐controlled analgesia (PCA) techniques are useful options in this subgroup.

While these recommendations will not review adjunctive pain medication use in detail, it is important to note that multiple studies in children support adjunctive therapies such as nonopioid pain relievers (acetaminophen, nonsteroidal ant inflammatory agents, gabapentin, tricyclic antidepressants), and/or regional anesthesia techniques to improve pain control for chronic pain patients undergoing surgery.^117^ In pediatric patients the use of multimodal analgesic treatment results in decreased episodes of substantial pain.^123^ In addition, nonpharmacological interventions such as acupuncture and massage therapy have been shown to be effective in ameliorating pain after surgery and decreasing chronic pain symptoms in children.^124^, ^125^ Alternative medications such as dexmedetomidine and low dose ketamine (1‐5 mcg/kg/h) have been successfully employed as adjuvant therapy in pediatric patients with severe chronic pain.^126^, ^127^ It is also worthwhile to consider the contributions of fear, anxiety, and depression to opioid consumption. These issues should be discussed transparently and treated with psychological and pharmacological interventions as indicated on a case by case basis.^117^

*Recommendation*: For pediatric patients with chronic pain who are maintained on opioids, continue established preoperative dosing during the perioperative period as a baseline. Acute postsurgical analgesia should be provided over and above the baseline opioids. Use of nonopioid analgesia is encouraged including regional analgesia techniques, alpha‐2 agonists, ketamine, acetaminophen, nonsteroidal anti‐inflammatory drugs, and neuropathic pain medications such as gabapentinoids or antidepressants.

*Strength of evidence*: C.

*Recommendation*: If the patient with chronic pain on chronic opioid therapy undergoes a procedure that is intended to remove the source of the patient's pain, they should be monitored for over sedation and should be discharged home with a defined opioid weaning plan. Opioid weaning in this case should be managed by a physician with special training or expertise in pain medicine. Limited supplies of opioids should be prescribed and refilled at frequent intervals that include face‐to‐face follow up visits.

*Strength of evidence*: C (based on extrapolation of adult data and expert consensus).

*Recommendation*: If a patient with chronic pain on opioid therapy undergoes surgery and the patient's underlying pain source was independent of the surgery, the patient's baseline pain should continue to be managed by the physician who had been doing so preoperatively. Opioid analgesics for the perioperative pain, if needed, should be prescribed in limited quantities consistent with the degree of physiologic trespass.

*Strength of evidence*: C (based on extrapolation of adult data and expert consensus).

### 10.2 Patients with central sensitization

Perioperative pain management for pediatric patients with central sensitization is largely based on consensus. In the pediatric population the cohort is largely made up of patients with fibromyalgia, former premature newborns with extensive procedure history from the intensive care nursery, those with cancer, and those with an extensive surgical history. These patients are characterized by increased sensitivity to painful or nonpainful stimuli. There is evidence in adults with fibromyalgia that they are at risk for increased acute and chronic postoperative pain.^128^, ^129^

*Recommendation*: For pain management of patients with central sensitization—strategies should be similar to those for pediatric pain patients on chronic opioids (use nonopioid analgesic techniques to the greatest extent possible). Opioids should be prescribed as needed. These patients benefit from the involvement of a pain physician for the purposes of assuring appropriate use and discontinuation of medications in a reasonable time frame.

*Strength of evidence*: C.

## 11 assessment of pain and analgesic efficacy

Topics covered in this section:

- How should we assess pain intensity?
- Is there evidence for pain behavior observation, assessing the response to treatment, postoperative recovery and function?

Pediatric anesthesiologists are routinely asked to assist in perioperative pain management, and there is wide agreement that appropriate assessment of pain is necessary in order to better guide care during the perioperative and postoperative period.^2^, ^130^, ^131^, ^132^ Unfortunately, there are insufficient data to support the beneficial effect of routine pain assessment on patient outcomes.^133^ Furthermore, observational data evaluating institutional practices demonstrate inconsistencies in the frequency and nature of pain assessment in many hospital settings.^134^, ^135^, ^136^, ^137^ Inappropriate assessment and/or misinterpretations of pain assessments may lead to under‐ or over‐use of analgesics and suboptimal or even harmful outcomes.^138^, ^139^, ^140^ Consensus documents addressing pain management in children recommend that assessment of a child's pain should be multi‐dimensional (location, nature, intensity), developmentally guided, and include the use of well‐established instruments (those with at least two peer reviewed studies by different teams that have established reliability and validity).^2^, ^130^, ^131^, ^132^ It is also recommended that pain assessments (including use and interpretation of pain intensity scores) be made in consideration of the unique situational context (eg, type and nature of surgery), treatments received, and psycho‐social factors.^141^, ^142^, ^143^

*Recommendation*: Regular pain assessments should be part of the perioperative care/treatment of pediatric patients who are receiving opioid medications. These assessments should be made using validated measures. The pain assessment should consider the unique circumstances of the child's psychological state and the extent of surgery.

*Strength of evidence*: B.

### 11.1 Self‐report measures

Observational studies demonstrate children greater than 4 years of age can reliably use self‐report instruments to describe and score pain intensity. However, children less than 8 years of age have exhibited difficulty distinguishing between sensory pain (eg, incisional pain, and distress or affect, ie fear),^132^, ^144^ and often exhibit scoring biases.^145^ Additionally, young children aged four to eight have been observed giving differing pain scores to different people during a painful procedure.^146^ There is insufficient evidence to know whether older children or adolescents exhibit scoring biases.

Systematic reviews and consensus panels have identified several self‐report measures that are considered well established in measuring pain intensity during procedures, after surgery, and for use in clinical analgesic trials.^147^, ^148^ These include (but are not limited to) the 0‐10 Numeric Rating Scale (NRS), Faces Revised Pain Scale (FRPS), and the color analog scale. It is widely recommended by consensus groups a self‐report of pain intensity be obtained whenever possible to assess and document the child's perceived level of pain intensity^130^, ^131^, ^149^ and that behavioral observation be used to complement self‐report, particularly when scores are inconsistent with the clinical presentation, contextual factors, or appear to be biased.^130^, ^140^

Several observational studies have reported average self‐reported numeric or faces pain intensity scores children associate with perceptions of pain severity and their need for analgesia (ie, in general, higher scores are more often associated with perceived need).^142^ However, wide variability has been observed in children's pain perceptions and reported population averages may not reflect the needs of the individual child. Pain intensity scores must therefore be interpreted in context. For instance, studies that based titration of opioid doses on specific pain scores (most commonly to achieve a score of less than four out of ten) have resulted in high rates of excessive sedation.^150^ Other, more global comfort and function outcomes (ability to take a deep breath and cough, get out of bed, etc), are more nebulous but better indicators of analgesic need compared to arbitrary target pain scores.

Recommendation: Pain intensity scores can be used to assess a child's perceived degree of discomfort. However, decisions to administer analgesics should take into consideration patient functional behaviors, situational factors such as pain source, self‐reported pain scores, parental observation, and other potential sources of distress, rather than arbitrarily selected pain score cutoffs.

*Strength of evidence*: B.

### 11.2 Behavioral observation

Behavioral observation instruments can be reliably used to document pain behaviors in children who cannot self‐report, including those younger than 4 years of age, infants, neonates, and children or adolescents with moderate to severe cognitive impairments. Observational studies have demonstrated behavioral observation instruments can be used reliably between providers to document pain behaviors.^149^, ^151^, ^152^ These measures include (but are not limited to):

- Behavior and posture‐related scales (Faces, Legs, Activity, Cry, Consolability [FLACC], Children's Hospital of Eastern Ontario Pain Scale [CHEOPS])
- Combined behavioral/physiologic parameter scales (Crying, Requires O_2_, Increased Vital Signs, Expression, Sleepless [CRIES])

Observational evidence also suggests behavioral pain scores are moderately consistent with nurses’ and parents’ global assessments when assessing pain in children who cannot self‐report. Observed pain behaviors correlate significantly but not consistently with children's self‐report.^149^

There is insufficient evidence that behavioral observation instruments can differentiate pain distress from other sources of distress such as physiologic compromise or fear.^152^, ^153^ Thus, consensus publications recommend behavioral observation measures be used to assess presence and intensity of pain in children who cannot self‐report, but also those scores be interpreted with caution and in consideration of the situational context and other factors.

*Recommendation*: Behavioral observation can be used to assess pain‐related distress in children. Directions to administer analgesics in children who cannot self‐report should take into consideration situational factors such as pain source, observational pain scores, and other potential sources of distress, rather than arbitrarily selected pain score cut‐offs.

*Strength of evidence*: B.

### 11.3 Analgesic responsiveness

Data from observational and clinical trials of analgesics have demonstrated that self‐report and behavioral observation pain intensity scores are sensitive in detecting pain relief after analgesic administration and over time (ie scores decrease significantly after administration).^154^, ^155^, ^156^, ^157^, ^158^, ^159^, ^160^ Observational studies have also reported “Mean Clinically Significant Differences” in that the average decrease in pain score reflects perceived pain relief for children as young as 6 and 8 years.^157^, ^161^ On average, a decrease of one point on the 0‐10 NRS is associated with a child's perception of pain relief. However, the data show wide variability and inconsistencies in reporting (eg some scores go up when the child reports relief and these are not stable across pain intensity ratings).

*Recommendation*: Changes in pain scores should be used in conjunction with other verbal/behavioral measures as indicators of pain relief and analgesic response (eg side effects) when making analgesic decisions.

*Strength of evidence*: B.

### 11.4 Physiologic measures

Although an acute pain insult can induce changes in physiologic vital sign parameters such as heart rate and blood pressure, observational data have shown that these changes are nonspecific and poor sensitivity pain indicators.^162^, ^163^ Recent data have demonstrated correlation between high frequency heart rate variability, known as the Analgesia Nociception Index (ANI), and observed FLACC scores in a small sample of children. The data suggest that an objective, physiologic, indicator of pain based on parasympathetic tone may be useful for children who cannot self‐report pain.^156^ Such data are relatively sparse, and the clinical usefulness of this tool remains to be proven.

*Recommendation*: Physiologic parameters should be used to assess the child's nociceptive response when it is not possible to assess pain with self‐report of behavioral measures (eg, when the child is sedated or is receiving neuromuscular blockers). It is recommended that other potential sources of physiologic distress (eg, emotional distress, hypovolemia, fever, hypercarbia) be considered and/or ruled out when making treatment decisions.

*Strength of evidence*: B.

### 11.5 Postoperative recovery and function

Consensus opinion supports assessment of the child's physical functioning when assessing recovery from pain and surgery, yet there is insufficient evidence regarding valid or reliable measures to assess children's physical function related to acute postoperative pain.^147^ Pain interference instruments categorize the degree to which pain hinders engagement with social, cognitive, emotional, physical, and recreational activities. Observational data support the reliability of parental functional assessment of the child's pain interference after surgery.^164^, ^165^ Pain interference also incorporates items that address ability to sleep and school attendance. These tools are available for adults and pediatric self‐reporters as well as a version where parents serve as proxy reporters.

*Recommendation*: A child's functional recovery should be assessed to inform treatment plans.

*Strength of evidence*: C.

### 11.6 Nature and location of pain

Observational studies have demonstrated children as young as 4 years can reliably identify a pain location (ie body map) that is consistent with clinical findings.^166^, ^167^ Additionally, observational data suggest that children as young as 8 years of age can describe the quality and nature of pain using word lists and scales.^168^

*Recommendation*: Assessing pain location is recommended to differentiate incisional pain from other potential sources of postoperative pain. The nature of pain should be assessed to assist in the differentiation of pain type.

*Strength of evidence*: B.

## 12 monitoring of patients on perioperative opioid therapy

Topics covered in this section:

- How should we utilize physiologic assessment of pain?
- Is there a strategy for monitoring sedation from opioids?
- What is the role of the institution in monitoring practice?

Because of variability in both efficacy and adverse effects of opioid analgesics among pediatric patients in the immediate postoperative time period monitoring (through technology and human observation) should include vital signs and level of sedation to assess adverse effects of opioids. The ASA, the Anesthesia Patient Safety Foundation (APSF), the Joint Commission, Centers for Medicare and Medicaid Services (CMS), and the American Pain Society (APS) have promulgated practice guidelines incorporating the recommendations for monitoring discussed below.^8^, ^169^, ^170^, ^171^, ^172^, ^173^

### 12.1 Physiologic monitoring

There are no prospective randomized trials investigating the minimum monitoring requirements concerning pediatric patients on PCA opioid infusions. Additionally, there are no clinical trials correlating levels of monitoring with significant adverse outcomes. In spite of this, a national survey of members of SPA, representing 252 institutions in the United States, showed 90% of pediatric anesthesiologists monitored patients on PCA with continuous pulse oximetry.^72^ Consensus documents and expert opinion advise basic monitoring of respiratory and cardiovascular status to recognize hypoventilation and apnea, particularly for those who are naïve to the drug and modality.^79^, ^172^, ^173^, ^174^, ^175^ Experts agree physiologic monitoring of pediatric patients receiving initial doses of parenteral opioids or opioids by PCA/NCA/PCA‐P (parent and proxy) and/or constant infusion should include respiratory rate (by plethysmography or direct observation), and continuous pulse oximetry. Although it is a sensitive measure of hypoventilation, capnography is not routinely recommended, however its use has been described.^176^, ^177^, ^178^, ^179^ To date, capnography has been shown to be largely impractical in children in the postoperative setting.^175^

Transcutaneous PaCO_2_ monitors have been evaluated in pediatric populations,^180^ including a combined transcutaneous oximeter/carbon dioxide monitor.^181^ Unfortunately, none of these have been evaluated in infants and children receiving opioid medications (specifically) in the postoperative setting.

New technologies, such as respiratory volume monitoring,^182^ and detection of expired gas moisture or temperature, have been described, but have not been tested or validated in children for monitoring opioid effects.

Although there are no studies to specifically differentiate the monitoring requirements for populations that are logically at increased risk for opioid associated respiratory depression, additional vigilance is recommended in such patients. These populations include:

1. Neonates (because of immaturity of respiratory control mechanisms as well as drug metabolism and clearance)
2. Patients with cerebral palsy (poor coordination of airway musculature, increased secretions)
3. Patients with neuromuscular diseases (weakness of muscles of respiration [chest wall] and airway musculature)
4. Patients with cognitive impairment (difficult assessment of pain, sedation, level of consciousness)
5. Patients with sleep disordered breathing and obstructive sleep apnea patients (related to anatomic abnormalities such as Pierre‐Robin, obesity, tonsil‐adenoid hypertrophy)
6. Patients receiving poly‐pharmacy (co‐administration of benzodiazepines and/or sedatives)^182^, ^183^
7. Patients who have had an increase in PRN bolus dose or PCA dose of opioid, or addition of basal infusion rate to PCA
8. Opioid naïve patients (particularly on the first postoperative night)
9. Patients on supplemental oxygen (which impairs the sensitivity and response time of pulse oximetry as a monitor for apnea/hypopnea)
10. Patients receiving neuraxial opioids.

Meta‐analysis of adult studies has shown the risk of respiratory depression to be similar between "as needed" bolus administration of opioid and PCA bolus administration.^184^

Recommendation: Physiologic monitoring of children receiving initial intravenous opioid treatment should include pulse oximetry for the first 24 hours unless the patient is awake and actively being observed. Continuous monitoring of respiratory rate and electrocardiogram should be considered in pediatric patients who are on oxygen, or who have risk factors for respiratory depression. Physiological monitoring may be suspended when the patient is alert, awake, and/or ambulating.

*Strength of evidence*: C.

*Recommendation*: Although not specifically addressed in the literature, the expert panel recommends frequent assessment of the quality, as well as the rate of respirations, should be performed by direct observation and recorded in the medical record. Increased frequency and intensity of these observations is recommended for children in high‐risk groups in addition to standard electronic monitoring.

*Strength of evidence*: C.

### 12.2 Monitoring patients with obstructive sleep apnea

Of all the populations outlined above, the most commonly encountered and well studied is the growing cohort of pediatric patients with sleep apnea. Brown et al^185^ prospectively studied the effects of pediatric recurrent desaturation events on sensitivity to the respiratory depressant effects of morphine in 20 children with obstructive sleep apnea. Having previously determined an increased risk in those with a nighttime oxygen saturation nadir of <85%,^106^ the patients were grouped into severe or moderate sleep apnea. Those with severe obstructive sleep apnea, as measured by low oxygen nadir, were more sensitive to morphine respiratory depression and required a lower total dose of morphine for pain management post tonsillectomy than those with mild sleep apnea. These patients should have their dose of opioid reduced by 50% to 67% in addition to requiring additional monitoring in the perioperative period when opioids are being administered.^186^ The increased risk of postoperative respiratory depression is also seen in adults with obstructive sleep apnea.^187^

Obesity is strongly associated with obstructive sleep apnea. Sleep apnea is also more common in certain pediatric populations including African Americans, Hispanic Americans, premature babies, toddlers who attend daycare, and children of lower socioeconomic status.^186^ Of these, African American and syndromic patients had the greatest risk with one study documenting an incidence 12.5 and 11 times that of the baseline Caucasian population.^188^, ^189^

Some adverse respiratory events may be traced to inaccurate dosing due to obesity. Specifically opiate dosing based on total body weight can result in dangerous respiratory depression.^190^, ^191^ Dosing should be based on ideal or lean body mass that can be calculated using one of many dosing scalers rather than rough estimates of appropriate weight dosing.^186^

*Recommendation*: Patients with obstructive sleep apnea, obesity (>95 percentile BMI), and recurrent nighttime oxygen desaturations are at higher risk for opioid induced respiratory depression. Opioid dosing should be based on ideal or lean body weight and the dose of opioid should be reduced by 50% to 67% for OSA patients. Additionally, extended respiratory monitoring is required when opioids are being administered to this population in the perioperative period.

*Strength of evidence*: B.

### 12.3 Monitoring level of sedation

Excessive sedation has been found to predict/precede opioid‐related respiratory depression and was observed prior to opioid‐related death/neurological injury in a majority of children.^182^ Therefore, patients receiving PCA or PCA‐P, with or without a basal infusion, should be monitored for their level of sedation. Sedation scales developed and validated for use in children undergoing procedural sedation, such as the Ramsey Scale or the University of Michigan Sedation Scale (UMSS), only include immediate response to commands or physical stimulation, may be less appropriate than a scale such as the Pasero Opioid Sedation Score (POSS). These scales require that the rater stimulate the patient and subsequently evaluate respiratory rate, depth, and pattern. Common sense requires respiratory evaluation in patients receiving opioid analgesia be done before stimulating the patient since measures after patient stimulation are inaccurate. The POSS grades the “drowsiness” of a patient based on the difficulty staying awake or being aroused during an examination. It also evaluates the ability of a patient to have a conversation with the rater and determines whether somnolence causes the patient to fall asleep during conversation.^192^, ^193^ This scale also includes suggested measures to respond to moderate (reduce opioid dose and observe) to excessive sedation (stop the opioid, administer naloxone). It is important to note there are no published data demonstrating the POSS is effective in preventing opioid‐related adverse outcomes in clinical pediatric settings. In spite of this, the inclusion of a measure such as the POSS is a logical tool to assess the opioid‐related sedation effect and is preferable to scales developed for procedural sedation, which also lack any evidence for effectiveness in this setting.

Recommendation: Patients receiving opioid analgesia perioperatively should have regular assessment of their level of sedation using a validated sedation score that evaluates level of alertness or mentation, rather than utilizing a procedural sedation scale. This rating should be part of the medical record along with measures of pain and physiological status.

Strength of Evidence: C.

### 12.4 Institutional surveillance/safety review for opioid‐related adverse events

Surveillance for opioid‐related adverse events has been recommended including regular review of rapid response team and/or code team calls and the need for naloxone administration. Publications of institutional review procedures of this nature provide information regarding risk for adverse events in both adults and children.^174^, ^194^, ^195^ Sophisticated filtering of physiologic monitoring data has been reported to detect prevalence of desaturation events in patients receiving opioids.^196^ Importantly, concerns have recently been raised about the contribution of “alarm fatigue” to failure to rescue with respect to opioid overdose.^197^

*Recommendation*: Review of code team calls or emergency response calls and delivery of emergent naloxone doses should be part of institutional efforts to critically evaluate and reduce preventable opioid‐related adverse events.

*Strength of evidence*: B.

## 13 opioid side effects in children

Topics covered in this section:

- Prevalence and management of pruritis, emesis, and ileus.

### 13.1 Pruritus

Opioid‐induced pruritus is a common problem, with reported incidences between 2% and 10% for intravenous morphine.^59^ The mechanism is not understood, but central mu‐receptor activity likely has an important influence. In the case of morphine, histamine and mast cell mediator release may affect peripheral receptors. Studies of treatment of opioid‐induced pruritus have been limited, particularly for intravenously administered opioids. Most notable is a randomized controlled trial of 184 children greater than 7 years old treated with PCA, given 50 mcg/kg of nalbuphine for itch. The authors found no benefit of nalbuphine.^198^ Nevertheless, a systematic review of 10 adult studies found overall benefit of nalbuphine for treatment of opioid‐induced pruritus.^199^

Naloxone has a mixed track record in pediatric perioperative care. West et al found no effect on itch in a randomized controlled pediatric trial of naloxone/morphine admixture in PCA.^200^ On the other hand, a randomized controlled trial with naloxone infusion given to children separately from the patient‐controlled intravenous morphine, in a dose finding study, Monitto and colleagues^201^ found naloxone to be effective for treatment of itch in over 90% of cases at a dose of 1 mcg/kg/h, without reversing analgesia or increasing required morphine doses. A randomized controlled trial of 46 pediatric patients using postoperative PCA found less pruritus with low‐dose (0.25 mcg/kg/h) naloxone infusion than placebo and that pain control was not adversely affected.^202^

*Recommendation*: Naloxone infusion is helpful in treating and possibly preventing opioid‐induced pruritus.

*Strength of evidence*: A.

*Recommendation*: Expert consensus supports the use of nalbuphine although there is conflicting evidence concerning its effectiveness at this time.

*Strength of evidence*: C.

### 13.2 Emesis

There are many studies of perioperative nausea and vomiting in children, but few evaluating the effect of opioid on the incidence of perioperative nausea and vomiting. Most of these have been studies in surgical populations with a high incidence of nausea and vomiting such as tonsillectomy and adenoidectomy or strabismus surgery. Perioperative opioids are known to increase postoperative nausea and vomiting when compared to nonsteroidal anti‐inflammatory drugs.^203^, ^204^ The magnitude of this effect varies with the type of surgery performed, anesthesia used, and patient population studied. Moiniche et al reported nausea and vomiting as a secondary endpoint in a systematic review of NSAIDs compared to opioid for tonsillectomy analgesia. The use of NSAIDs significantly improved nausea and vomiting when compared to opioids. This finding is consistent in several other studies of perioperative opioid use in children.^205^, ^206^, ^207^, ^208^ Conversely, in a prospective, randomized controlled trial of children given a propofol‐based anesthetic and dexamethasone for nausea and vomiting prophylaxis, Keidan et al compared ketorolac to fentanyl^209^ and found no appreciable difference in postoperative nausea and vomiting in children (baseline rate was much lower). Double‐blind, randomized, controlled trials show that the choice of anesthetic technique, the surgical procedure, and pharmacological prophylaxis affect the incidence of postoperative nausea and vomiting specifically related to intraoperative opioid use^210^, ^211^, ^212^, ^213^, ^214^, ^215^ as do lower morphine doses.^216^ Eberhart et al^217^ evaluated the contribution of opioids to perioperative nausea/vomiting in a meta‐analysis of propofol vs inhaled agents for maintenance of anesthesia. This group did not find that intraoperative opioids increased the risk of postoperative nausea and vomiting. Another systematic review of 13 randomized controlled pediatric trials of morphine compared to placebo or active control found that morphine had a significantly greater incidence of nausea than the active controls, including nerve blocks, tramadol, buprenorphine, and ketorolac.^203^

Similarly in some instances the same studies as mentioned for investigation of pruritus, the use of low‐dose continuous naloxone infusion has been studied for the prevention of nausea and vomiting. A randomized controlled trial of pediatric patients using postoperative PCA found less nausea with 0.25 mcg/kg/h naloxone infusion when compared to placebo. A dose finding study involving 59 pediatric patients found that infusion rates greater than or equal to 1 mcg/kg/h led to significantly less nausea than lower infusion rates.^218^ As mentioned previously, when used in low doses, naloxone has not been shown to significantly affect pain control or opioid usage.^202^, ^218^ Several other medications such as transdermal scopolamine,^219^ 5‐HT3 receptor antagonists (ie tropisetron,^220^ ondansetron,^203^ ramosetron^203^), and dixyrazine^221^ have also been shown to decrease the incidence of nausea and vomiting for postsurgical patients utilizing PCA opioids for analgesia. Each of these agents has a small risk of causing other side effects. Agents with anticholinergic and antihistaminic actions contribute to postoperative delirium, sedation, and bowel and bladder dysfunction. Nalbuphine has been found to be ineffective in pediatric patients for PCA‐related nausea and vomiting.^222^

*Recommendation*: Opioid use should be minimized where possible to decrease the incidence of nausea and vomiting (see section on Impact of Adjunctive Medications on Opioid Dosing and Side Effects for specific medication recommendations).

*Strength of evidence*: A.

*Recommendation*: Naloxone infusion should be considered for intravenous opioid therapy for the prevention or treatment of nausea and vomiting.

*Strength of evidence*: A.

*Recommendation*: It is reasonable to consider common anti‐emetic medications for the treatment or prevention of nausea and vomiting while on intravenous opioid therapy. Preference should be for nonsedating medications.

*Strength of evidence*: A.

### 13.3 Perioperative opioids and ileus

A common complication of opioids in both the acute and chronic care setting is slowing of bowel motility and specifically constipation or ileus. In the acute care setting, this can result in significant morbidity and delayed discharge. Postoperative opioid‐related decreased bowel motility or ileus is well documented in the adult population^223^, ^224^ and is importantly associated with readmission in children.^200^ Treatment and incidence of opioid‐induced ileus has only minimally been explored in the literature in infants and children.^225^, ^226^ Contrary to adult studies, methylnaltrexone appears to be helpful in children.^225^, ^226^, ^227^, ^228^

*Recommendation*: Postoperative opioid‐induced ileus may be improved with methylnaltrexone in infants and children.

*Strength of evidence*: B.

## 14 SUMMARY

The role of opioids in the treatment of pediatric perioperative pain is evolving, and professional as well as public concern about their appropriate application is currently at a high point. In spite of these concerns, opioids remain a part of the perioperative pain treatment armamentarium. These recommendations have been formulated as a guide to reinforce current concepts of safe and appropriate use of these potent medications. We do not present them as a comprehensive guide for all patient encounters, but rather as a specific set of recommendations for practice involving specific situations where opioids are employed. To the greatest extent possible we have utilized published evidence to formulate these recommendations however it is clear that many of these recommendations lack sufficient evidence to make firm, evidence‐based conclusions. We hope that researchers involved in pediatric pain management will utilize some of the discussions and conclusions as a guide for future clinical trials and outcome analysis. The taskforce involved in these recommendations will update them every 2 years and disseminate the updated recommendations through the SPA Website (http://www.pedsanesthesia.org) as well as peer reviewed publication.

## DISCLOSURES

Joseph P. Cravero is a Section Editor at Pediatric Anesthesia; Lynne Maxwell is an Associate Editor at Pediatric Anesthesia; Terri Voepel‐Lewis is an Associate Editor at Pediatric Anesthesia.

## ETHICAL APPROVAL

No IRB approval was required or obtained.

## APPENDIX 1

### 1.1

**Table A1:** Database searches for evidence for this document

The databases/search engines utilized for this set of recommendations included PubMed, Medline, Web of Science, EMBASE, Google Scholar, National Guideline Clearinghouse. Only English language papers were included. In many cases thousands of papers were generated. The author responsible for each section reviewed papers for relevance to the specific topics that would be covered and graded the evidence. The search results were then made available for review to the entire group of authors:
**Age‐related effects section**
*Opioids AND pediatrics AND (pharmacokinetics OR pharmacodynamics)*
((“analgesics, opioid”[mesh] OR opioid[tiab] OR opioids[tiab]) AND (pediatrics[mesh] OR pediatric[tiab] OR pediatrics[tiab]) AND ((pharmacokinetics[mesh] OR pharmacokinetics[tiab]) OR pharmacodynamics[tiab]))
*Opioids AND pediatrics AND respiratory depression*
((“analgesics, opioid”[mesh] OR opioid[tiab] OR opioids[tiab]) AND (pediatrics[mesh] OR pediatric[tiab] OR pediatrics[tiab]) AND (“respiratory insufficiency”[mesh] OR “respiratory insufficiency”[tiab] OR “respiratory depression”[tiab] OR “ventilator depression”[tiab]))
*Chest wall rigidity*
(((“thoracic wall”[mesh] OR “thoracic wall”[tiab] OR “chest wall”[tiab]) AND (“muscle rigidity”[mesh] OR rigidity[tiab])) OR (“chest wall rigidity”[tiab] OR “thoracic wall rigidity”[tiab]))
*Opioids AND metabolism*
((“analgesics, opioid”[mesh] OR opioid[tiab] OR opioids[tiab]) AND (metabolism[mesh] OR metabolism[tiab]))
*Neonates AND opioids AND pharmacokinetics*
((“infant, newborn”[mesh] OR infant[tiab] OR infants[tiab] OR newborn[tiab] OR newborns[tiab] OR neonate[tiab] OR neonates[tiab]) AND (“analgesics, opioid”[mesh] OR opioid[tiab] OR opioids[tiab]) AND (pharmacokinetics[mesh] OR pharmacokinetics[tiab]))
*Neonates AND opioids AND pharmacodynamics*
((“infant, newborn”[mesh] OR infant[tiab] OR infants[tiab] OR newborn[tiab] OR newborns[tiab] OR neonate[tiab] OR neonates[tiab]) AND (“analgesics, opioid”[mesh] OR opioid[tiab] OR opioids[tiab]) AND (pharmacodynamics[tiab]))
*Neonates AND fentanyl*
((“infant, newborn”[mesh] OR infant[tiab] OR infants[tiab] OR newborn[tiab] OR newborns[tiab] OR neonate[tiab] OR neonates[tiab]) AND (fentanyl[mesh] OR fentanyl[tiab] OR phentanyl[tiab] OR fentanest[tiab] OR sublimaze[tiab] OR duragesic[tiab] OR durogesic[tiab] OR fentora[tiab]))
*Neonates AND morphine*
((“infant, newborn”[mesh] OR infant[tiab] OR infants[tiab] OR newborn[tiab] OR newborns[tiab] OR neonate[tiab] OR neonates[tiab]) AND (morphine[mesh] OR morphine[tiab] OR contin[tiab] OR oramorph[tiab] OR duramorph[tiab]))
*Neonates AND sufentanil*
((“infant, newborn”[mesh] OR infant[tiab] OR infants[tiab] OR newborn[tiab] OR newborns[tiab] OR neonate[tiab] OR neonates[tiab]) AND (sufentanil[mesh] OR sufentanil[tiab] OR sulfentanyl[tiab] OR sulfentanil[tiab] OR sufenta[tiab] OR sufentanilratiopharm[tiab]))
*Neonates AND remifentanil*
((“infant, newborn”[mesh] OR infant[tiab] OR infants[tiab] OR newborn[tiab] OR newborns[tiab] OR neonate[tiab] OR neonates[tiab]) AND (remifentanil[Supplementary Concept] OR remifentanil[tiab] OR ultiva[tiab]))
**PCA section**
*Analgesia*
(analgesia[mesh] OR analgesia[tiab] OR analgesics[mesh] OR analgesics[tiab] OR analgesic[tiab])
*Patient‐controlled AND analgesia*
((analgesia[mesh] OR analgesia[tiab] OR analgesics[mesh] OR analgesics[tiab] OR analgesic[tiab]) AND “patient controlled”[tiab])
*Patient‐controlled AND analgesia AND (child OR pediatrics)*
(analgesia[mesh] OR analgesia[tiab] OR analgesics[mesh] OR analgesics[tiab] OR analgesic[tiab]) AND “patient controlled”[tiab] AND ((pediatrics[mesh] OR pediatric[tiab] OR pediatrics[tiab]) OR (child[mesh] OR child[tiab] OR children[tiab]))
**Monitoring section**
(“monitoring, physiologic”[mesh] OR “drug monitoring”[mesh] OR “drug monitoring”[tiab] OR “monitoring”[tiab])
*Opioid administration*
(“analgesics, opioid/administration”[mesh] OR ((“administration, intravenous”[mesh] OR “drug therapy”[mesh] OR administration[tiab]) AND (“analgesics, opioid”[mesh] OR opioid[tiab] OR opioids[tiab])))
*Inpatient*
(Inpatients[mesh] OR inpatients[tiab] OR inpatient[tiab])
*Clinical practice guidelines*
(“practice guidelines as topic”[mesh] OR guideline[publication type] OR guidelines[tiab] OR guideline[tiab])
*Patient‐controlled analgesia*
((analgesia[mesh] OR analgesia[tiab] OR analgesics[mesh] OR analgesics[tiab] OR analgesic[tiab]) AND (“patient controlled”[tiab] OR “patient controlled analgesia”[tiab]))
*PCA*
((analgesia[mesh] OR analgesia[tiab] OR analgesics[mesh] OR analgesics[tiab] OR analgesic[tiab]) AND (“patient controlled”[tiab] OR “patient controlled analgesia”[tiab] OR PCA[tiab]))
*Pediatric*
(pediatrics[mesh] OR pediatric[tiab] OR pediatrics[tiab])
*Parent*
(parents[mesh] OR parents[tiab] OR parent[tiab])
*Nurse*
(nurses[mesh] OR nursing[mesh] OR nurses[tiab] OR nurse[tiab])
*PCA by proxy*
((analgesia[mesh] OR analgesia[tiab] OR analgesics[mesh] OR analgesics[tiab] OR analgesic[tiab]) AND (“patient controlled”[tiab] OR “patient controlled analgesia”[tiab] OR PCA[tiab]) AND (proxy[mesh] OR proxy[tiab]))
*Critical events in inpatient receiving opioid*
((“medical errors”[mesh] OR “medical errors”[tiab] OR “medical error”[tiab] OR “medical mistakes”[tiab] OR “medical mistake”[tiab] OR “wrong procedure errors”[tiab] OR “wrong procedure error”[tiab] OR “critical medical incidents”[tiab] OR “critical medical incident”[tiab] OR “critical incidents”[tiab] OR “critical incident”[tiab] OR “never events”[tiab] OR “never event”[tiab] OR “critical events”[tiab] OR “critical event”[tiab]) AND (Inpatients[mesh] OR inpatients[tiab] OR inpatient[tiab]) AND (“analgesics, opioid”[mesh] OR opioid[tiab] OR opioids[tiab]))
*Critical event alerts networked monitoring*
((“medical errors”[mesh] OR “medical errors”[tiab] OR “medical error”[tiab] OR “medical mistakes”[tiab] OR “medical mistake”[tiab] OR “wrong procedure errors”[tiab] OR “wrong procedure error”[tiab] OR “critical medical incidents”[tiab] OR “critical medical incident”[tiab] OR “critical incidents”[tiab] OR “critical incident”[tiab] OR “never events”[tiab] OR “never event”[tiab] OR “critical events”[tiab] OR “critical event”[tiab]) AND (“monitoring, physiologic”[mesh] OR “drug monitoring”[mesh] OR “drug monitoring”[tiab] OR “event monitoring”[tiab] OR “monitoring”[tiab]))
*Hospital surveillance for critical events*
((“medical errors”[mesh] OR “medical errors”[tiab] OR “medical error”[tiab] OR “medical mistakes”[tiab] OR “medical mistake”[tiab] OR “wrong procedure errors”[tiab] OR “wrong procedure error”[tiab] OR “critical medical incidents”[tiab] OR “critical medical incident”[tiab] OR “critical incidents”[tiab] OR “critical incident”[tiab] OR “never events”[tiab] OR “never event”[tiab] OR “critical events”[tiab] OR “critical event”[tiab]) AND (“monitoring, physiologic”[mesh] OR monitoring[tiab] “event monitoring”[tiab] OR surveillance[tiab]))
*Respiratory monitoring technologies*
((“monitoring, physiologic”[mesh] OR “drug monitoring”[mesh] OR “drug monitoring”[tiab] OR “monitoring”[tiab]) AND (“respiratory rate”[mesh] OR “respiratory rate”[tiab]) AND (technology[mesh] OR technology[tiab]))
*Adult and pediatric*
((adult[mesh] OR adult[tiab]) AND (pediatrics[mesh] OR pediatric[tiab] OR pediatrics[tiab]))
*Smart systems*
(“smart systems”[tiab] OR “smart system”[tiab] OR (smart[tiab] AND (systems[tiab] OR system[tiab])))
**Monitoring pain levels section**
*Opioid*
(“analgesics, opioid”[mesh] OR opioid[tiab] OR opioids[tiab]))
*Pain assessment*
(“pain measurement”[mesh] OR “pain measurement”[tiab] OR “pain measurements”[tiab] OR “pain assessment”[tiab] OR “pain assessments”[tiab] OR “analgesia test”[tiab] OR “analgesia tests”[tiab] OR “nociception test”[tiab] OR “nociception tests”[tiab] OR “pain questionnaire”[tiab] OR “pain questionnaires”[tiab] OR “pain scale”[tiab]
OR “pain scales”[tiab] OR “formalin test”[tiab] OR “formalin test”[tiab] OR “pain test”[tiab] OR “pain tests”[tiab])
*Children*
(child[mesh] OR child[tiab] OR children[tiab])
*Human*
(humans[mesh] OR humans[tiab] OR human[tiab])
**Side effects section**
*Infant*
(infant[mesh] OR infant[tiab] OR infants[tiab])
*Neonate*
(“infant, newborn”[mesh] OR infant[tiab] OR infants[tiab] OR newborn[tiab] OR newborns[tiab] OR neonate[tiab] OR neonates[tiab])
*Child*
(child[mesh] OR child[tiab] OR children[tiab])
*Adolescent/teen*
(adolescent[mesh] OR adolescent[tiab] OR adolescents[tiab] OR teen[tiab] OR teens[tiab] OR teenager[tiab] OR teenagers[tiab] OR youth[tiab] OR youths[tiab])
*Toddler pediatric AND opioid or opiate*
(((toddler[tiab] OR toddlers[tiab]) AND (pediatrics[mesh] OR pediatric[tiab] OR pediatrics[tiab])) AND ((“analgesics, opioid”[mesh] OR opioid[tiab] OR opioids[tiab]) OR (“opiate alkaloids”[mesh] OR “opiate alkaloids”tiab] OR “opiate alkaloid”[tiab] OR “alkaloid opiates”[tiab] OR “alkaloid opiate”[tiab] OR opiates[tiab] OR opiate[tiab])))
*Pruritis*
(pruritis[mesh] OR pruritis[tiab] OR itching[tiab])
*Urinary retention*
(“urinary retention”[mesh] OR “urinary retention”[tiab])
*Constipation*
(constipation[mesh] OR constipation[tiab] OR dyschezia[tiab] OR “colonic intertia”[tiab])
*Drug‐related side effects*
(“drug‐related side effects and adverse reactions”[mesh] OR (“drug‐related side effects and adverse reactions”[tiab] OR “side effects of drugs”[tiab] OR “drug side effects”[tiab] “drug side effect”[tiab] OR “adverse drug reactions”[tiab] OR “adverse drug reaction”[tiab] OR “adverse drug events”[tiab] OR “adverse drug event”[tiab] OR “drug toxicity”[tiab] OR “drug toxicities”[tiab])
*Paralytic ileus*
(“intestinal pseudo‐obstruction”[mesh] OR “intestinal pseudo‐obstruction”[tiab] OR “intestinal pseudo‐obstructions”[tiab] OR “paralytic ileus”[tiab] OR “visceral myopathy”[tiab] OR “visceral myopathies”[tiab])
*Ileus*
(ileus[mesh] OR ileus[tiab])
*Perioperative*
(“perioperative period”[mesh] OR “perioperative period”[tiab] OR “perioperative”[tiab])
*Methylnaltrexone OR alvimopan*
((methylnaltrexone [Supplementary Concept] OR methylnaltrexone[tiab] OR “quaternary ammonium naltrexone”[tiab] OR “naltrexone methylbromide”[tiab] OR relistor[tiab] OR “naltrexonium methiodide”[tiab]) OR (alvimopan[Supplementary Concept] OR alvimopan[tiab] OR entereg[tiab]))
**Opioid treatment of the patient with chronic pain scheduled for major surgery section**
*Chronic pain and chronic opioids*
(“narcotic dependence” [mesh] or “opiate dependence”[mesh] or “chronic narcotic”[mesh} AND “Anesthesia/ or anesthesia surgery” [tiab} OR “General Surgery/ Perioperative Nursing/ or perioperative” [tiab] or “Perioperative Care/ or Perioperative Period postoperative” [tiab] or “Postoperative Care” OR “Pain, Postoperative/ or Postoperative Complications” or “Postoperative Period” [tiab])
*Central sensitization*
(“fibromyalgia” OR “Fibromyalgia”[mesh] AND “methadone” [mesh] OR “Methadone/ buprenorphine” [mesh] OR “Buprenorphine Opiate Substitution Treatment”[mesh] OR “opioid maintenance”[mesh] or S”ubstance Withdrawal Syndrome”[mesh])

Abbreviation: pca, patient‐controlled analgesia.
